# The Impact of Prematurity on Morbidity and Mortality in Newborns with Dextro-transposition of the Great Arteries

**DOI:** 10.1007/s00246-021-02734-7

**Published:** 2021-09-24

**Authors:** Vinzenz Boos, Christoph Bührer, Mi-Young Cho, Joachim Photiadis, Felix Berger

**Affiliations:** 1 Department of Congenital Heart Disease/Pediatric Cardiology, German Heart Center Berlin, Augustenburger Platz 1, 13353 Berlin, Germany; 2 Department of Neonatology, Charité - Universitätsmedizin Berlin, Berlin, Germany; 3 Department of Neonatology, Hospital Zollikerberg, Zollikerberg, Switzerland; 4 Department of Congenital Heart Surgery / Pediatric Heart Surgery, German Heart Center Berlin, Berlin, Germany; 5 German Center for Cardiovascular Research (DZHK), Congenital Heart Diseases, Partner Site Berlin, Berlin, Germany

**Keywords:** Arterial switch operation, Mortality, Prematurity, Transposition of the great arteries

## Abstract

Prematurity is a risk factor for adverse outcomes after arterial switch operation in newborns with d-TGA (d-TGA). In this study, we sought to investigate the impact of prematurity on postnatal and perioperative clinical management, morbidity, and mortality during hospitalization in neonates with simple and complex d-TGA who received arterial switch operation (ASO). Monocentric retrospective analysis of 100 newborns with d-TGA. Thirteen infants (13.0%) were born premature. Preterm infants required significantly more frequent mechanical ventilation in the delivery room (69.2% vs. 34.5%, *p* = 0.030) and during the preoperative course (76.9% vs. 37.9%, *p* = 0.014). Need for inotropic support (30.8% vs. 8.0%, *p* = 0.035) and red blood cell transfusions (46.2% vs. 10.3%, *p* = 0.004) was likewise increased. Preoperative mortality (23.1% vs 0.0%, *p* = 0.002) was significantly increased in preterm infants, with necrotizing enterocolitis as cause of death in two of three infants. In contrast, mortality during and after surgery did not differ significantly between the two groups. Cardiopulmonary bypass times were similar in both groups (median 275 vs. 263 min, *p* = 0.322). After ASO, arterial lactate (34.5 vs. 21.5 mg/dL, *p* = 0.007), duration of mechanical ventilation (median 175 vs. 106 h, *p* = 0.038), and venous thrombosis (40.0% vs. 4.7%, *p* = 0.004) were increased in preterm, as compared to term infants. Gestational age (adjusted unit odds ratio 0.383, 95% confidence interval 0.179–0.821, *p* = 0.014) was independently associated with mortality. Prematurity is associated with increased perioperative morbidity and increased preoperative mortality in d-TGA patients.

## Introduction

Congenital heart disease (CHD) and prematurity are two of the leading causes of perinatal mortality [1]. Premature infants with CHD have a particularly increased risk for morbidity and mortality [2]. Infants with complex cardiovascular malformations are twice as likely to be born premature. However, dextro-transposition of the great arteries (d-TGA) has one of the lowest defect-specific percentages of premature births of about 8–11%, comparable to the risk of prematurity in infants without CHD [1, 3].

d-TGA requires anatomical repair in the neonatal period. The arterial switch operation (ASO) is the surgical treatment of choice with low perioperative mortality [4, 5]. ASO is typically performed shortly after birth, and delay of surgery is associated with increased morbidity and mortality [6]. Prematurity has been described as risk factor for perioperative morbidity, increased length of postoperative hospitalization, and in-hospital mortality in newborns with d-TGA [7–10]. In addition, premature infants with d-TGA are likely to be of low birth weight. Although ASO can be performed in neonates with a weight below 2000 g, the rates for perioperative morbidities and mortality are increased compared to infants weighing more than 2000 g [11, 12].

The management of premature infants with d-TGA, and complex CHD in general, presents numerous challenges related to inadequate maturation of multiple organ systems. They include the postnatal transition, hemodynamic and respiratory stabilization, including definition of target values for oxygenation and (permissive) hypercapnia, optimal nutrition, fluid and electrolyte management, prevention of infections and gastrointestinal, neurological, pulmonary, and renal complications. Furthermore, optimal timing for cardiac surgery is crucial to optimize perioperative outcomes [12, 13].

There are limited data on perioperative, particularly postnatal, preoperative morbidities, and mortality in premature infants with d-TGA. Previous studies investigated more heterogeneous cohorts of newborns with various types of complex CHD or focused mainly on operative outcomes [7, 14, 15]. The purpose of this study was to assess the impact of prematurity on postnatal, mainly preoperative medical treatment, as well as morbidity and mortality during hospitalization in infants with d-TGA in a single institutional cohort.

## Patients and Methods

We included all newborns diagnosed with d-TGA, both with and without associated cardiac lesions such as septal defects or coarctation, who were born between January 2013 and December 2017, and whose planned surgical therapy was ASO. All perinatal and perioperative data were retrieved from medical files and operative notes. The study received approval by the institutional review board (# EA2/069/17). The study cohort was divided into two groups for comparison: infants who were born before 37 completed weeks of gestational age were assigned to the “premature group,” whereas term born infants were assigned to the control group.

For infants with prenatal diagnosis of d-TGA, neonatologists and pediatric cardiologists were present in the delivery room. Infants who were born in external hospitals without prenatal diagnosis of d-TGA were transferred to our units shortly after diagnosis. All patients who survived the preoperative period subsequently received ASO in our institution, including the repair of associated lesions, if present.

The following data were analyzed for each patient: (1) demographic data, including sex, gestational age, birth weight, and multiple pregnancies; (2) obstetric data, including maternal age, administration of antenatal steroids, and premature rupture of membranes; (3) data on postnatal adaptation, including umbilical arterial blood pH, Apgar scores, and neonatal management in the delivery room; (4) preoperative management, such as inotropic support, mechanical ventilation, and co-morbidities, including modified Bell stage IIa or higher necrotizing enterocolitis (NEC), intracranial hemorrhage, suspected infection, based on clinical signs and laboratory tests, and preoperative mortality; (5) cardiac anatomy and coronary artery pattern; (6) operative data, including age and weight at surgery, transfusion during the course of cardiopulmonary bypass (CPB), and intraoperative death; (7) details on postoperative treatment, such as extracorporeal membrane oxygenation (ECMO), cardiac arrhythmia requiring medication or pacing, duration of postoperative ventilation, postoperative co-morbidities, including NEC and culture-proven sepsis, and postoperative mortality; (8) duration of hospitalization and overall mortality.

All missing information are noted in the results tables. All statistical analyses were conducted using R studio v1.4.1106 (RStudio, Boston, MA) with R v4.0.4 (The R Foundation for Statistical Computing, Vienna, Austria). Descriptive data for continuous variables are presented as median and interquartile range. Categorical variables are presented as relative frequencies. Comparison between groups (premature vs. term born) was chosen, as the relatively small number of patients in this single-center study impedes analysis by gestational age. Differences for continuous variables between the two groups were analyzed using Wilcoxon rank sum test, and Fisher’s exact test was used for comparison of dichotomous variables. Multivariable analysis was conducted on variables that reached significance with *p* value ≤ 0.1 in univariate analysis, using a logistic regression model, with demographics, maternal, and anatomic variables as input variables and mortality as output variable. Odds ratios (OR) are presented with 95% confidence interval (CI) and Nagelkerke’s R-squared is reported (goodness-of-fit). A *p* value < 0.05 was considered statistically significant.

## Results

Inclusion criteria were met by 100 neonates. 13 of these (13%) were premature born infants, all remaining infants were born at term (37 0/7 to 41 6/7 weeks of gestational age). No infant was born post term (42 0/7 weeks of gestational age and older). The only outborn patient in the cohort, a preterm infant, was diagnosed with d-TGA on the first day of life and transferred on the second day of life. Premature infants had a lower birth weight (2300 vs. 3280 g, *p* < 0.001) and received more often antenatal steroids (38.5% vs. 1.1%, *p* < 0.001). We observed a trend toward more Cesarean deliveries in preterm infants (61.5% vs. 32.2%, *p* = 0.061) and prematures required significantly more frequent mechanical ventilation in the delivery room (69.2% vs. 34.5%, *p* = 0.030), as compared to term babies (Table 1).

**Table 1:** Demographic characteristics, obstetric data, and postnatal adaptation

Variable	Premature infants (*n* = 13)	Term neonates (*n* = 87)	*p* value
	Patients (%) or median (IQR)	Patients (%) or median (IQR)	
*Demographics*			
Sex, male	10 (76.9)	55 (63.2)	0.534
Gestational age (weeks)	35.1 (33.3–36.3)	39.1 (38.4–40.0)	< 0.001
Birth weight (g)	2300 (1850–2515)	3280 (3008–3565)	< 0.001
Multiple gestation	3 (23.1)	2 (2.3)	0.015
Genetic abnormality	0 (0.0)	1 (1.1)	1.000
Extracardiac malformation	0 (0.0)	8 (9.2)	0.592
Prenatal diagnosis of CHD	11 (84.6)	75 (86.2)	1.000
Age at postnatal diagnosis of CHD (days)^a^	1 (1–1)	1 (1–2)	0.331
*Obstetric data*			
Maternal age (years)	31 (28–31)	30 (26–35)	0.565
Antenatal steroids	5 (38.5)	1 (1.1)	< 0.001
Premature rupture of membranes	1 (7.7)	1 (1.1)	0.244
Cesarean delivery	8 (61.5)	28 (32.2)	0.061
*Postnatal adaptation*			
Umbilical arterial blood pH^a^	7.27 (7.24–7.30)	7.28 (7.25–7.32)	0.591
Apgar at 5 min^a^	8 (7–9)	9 (8–9)	0.257
Apgar at 10 min^a^	8.5 (8–9)	9 (8–9)	0.416
Meconium aspiration	0 (0.0)	0 (0.0)	N/A
Respiratory support in DR	10 (76.9)	54 (62.1)	0.367
Mechanical ventilation in DR	9 (69.2)	30 (34.5)	0.030
Cardiopulmonary resuscitation in DR	1 (7.7)	4 (4.6)	0.509

*CHD* congenital heart disease, *DR* delivery room, *IQR* interquartile range, *N/A* not applicable

^a^The following information (no. of infants) was missing: Umbilical arterial blood pH  1, Apgar score at 5 min = 1, and Apgar score at 10 min = 1 in the “premature infants” group; age at postnatal diagnosis of CHD = 1 in the control group

Cardiac anatomy and coronary artery pattern were similarly distributed in premature and term infants. The majority of infants had isolated d-TGA and usual coronary artery pattern (1L,Cx;2R) (Table 2).

**Table 2:** Cardiac anatomy and coronary artery pattern

Variable	Premature infants (*n* = 13)	Term neonates (*n* = 87)	*p* value
	Patients (%)	Patients (%)	
*Cardiac anatomy*			
Isolated d-TGA (Intact ventricular septum)	9 (69.2)	55 (63.2)	0.765
d-TGA with associated lesions	4 (30.8)	32 (36.8)	0.765
VSD	3 (23.1)	20 (23.0)	1.000
VSD + DORV	0 (0.0)	1 (1.1)	1.000
VSD + LVOTO	0 (0.0)	1 (1.1)	1.000
CoA	0 (0.0)	1 (1.1)	1.000
CoA + VSD	1 (7.7)	7 (8.0)	1.000
CoA + VSD + LVOTO	0 (0.0)	1 (1.1)	1.000
CoA + VSD + pulmonary stenosis	0 (0.0)	1 (1.1)	1.000
Restrictive atrial septum^a^	1 (7.7)	2 (2.3)	0.345

Coronary artery pattern^b^	Premature infants (*n* = 10)	Term neonates (*n* = 87)	
1L,Cx;2R (usual)	8 (80.0)	56 (64.4)	0.487
Coronary artery pattern other than usual	2 (20.0)	31 (35.6)	0.487
*Two coronary Ostia*			
1L;2R,Cx (Cx from RCA)	2 (20.0)	14 (16.1)	0.668
1R;2L,Cx (inverted)	0 (0.0)	4 (4.6)	1.000
1L,R;2Cx (inverted RCA and Cx)	0 (0.0)	3 (3.4)	1.000
Other coronary anomaly (two ostia)	0 (0.0)	5 (5.7)	1.000
Two coronary ostia (total)	10 (100.0)	82 (94.3)	1.000
*Single coronary ostium*			
2L,Cx,R (single RCA)	0 (0.0)	4 (4.6)	1.000
1L,Cx,R (single LCA)	0 (0.0)	1 (1.1)	1.000
Single coronary ostium (total)	0 (0.0)	5 (5.7)	1.000
Intramural coronary artery			
Intramural LCA	1 (10.0)	1 (1.1)	0.197
Intramural RCA	0 (0.0)	1 (1.1)	1.000
Intramural coronary artery (total)	1 (10.0)	2 (2.3)	0.281

*CoA* coarctation of the aorta, d*-TGA* dextro-transposition of the great arteries, *DORV* double-outlet right ventricle, *LCA* left coronary artery, *LVOTO* left ventricular outflow tract obstruction, *RCA* right coronary artery, *VSD* ventricular septal defect

^a^One premature infant with VSD and two term born infants with isolated d-TGA

^b^For those who received cardiac surgery (10 and 87, respectively)

All infants received intravenous Prostaglandin E1 to maintain ductal patency. The rate of balloon atrial septostomy was equally high in both groups (76.9% vs. 82.8%, *p* = 0.699) and performed at median day 1 of life. More co-morbidities were observed in premature infants during the preoperative stay on neonatal ICU. They required more intensive care measures, such as more frequent inotropic support (30.8% vs. 8.0%, *p* = 0.035), mechanical ventilation (76.9% vs. 37.9%, *p* = 0.014), and red blood cell (RBC) transfusions (46.2% vs. 10.3%, *p* = 0.004). One premature infant born at 36 1/7 weeks with suspected coarctation received diagnostic cardiac catheterization on day 7, followed by coarctation repair on day 11. Due to failure to thrive, ASO was performed on day 59 in this neonate. Three prematures died before receiving ASO. One infant born at 30 6/7 weeks developed extended NEC with multiple perforations in small intestine and colon on day 9 and died two days later. A premature born at 31 4/7 weeks developed mesenteric artery thrombosis and NEC on day 14 and died on day 20, with large parts of intestine being gangrenous. One infant born at 32 6/7 weeks, who received an interventional placement of a stent in the foramen ovale on day 40 due to decreasing oxygen saturations, required atrial septectomy on day 63 due to hypoxemia. This infant died on day 83 because of severe pulmonary hypertensive crises. Preoperative mortality was significantly increased in premature infants with d-TGA, as compared to term patients (23.1% vs. 0.0%, *p* = 0.002). No patient in the entire cohort received inotropic support or mechanical ventilation when transferred to the operating room for ASO (Table 3).

**Table 3:** Clinical management and co-morbidities prior to arterial switch operation

Variable	Premature infants (*n* = 13)	Term neonates (*n* = 87)	*p* value
	Patients (%) or median (IQR)	Patients (%) or median (IQR)	
Temperature at NICU admission (°C)^a^	36.7 (36.4–37.3)	36.8 (36.4–37.2)	0.642
Prostaglandin E1 infusion	13 (100.0)	87 (100.0)	N/A
Duration of prostaglandin E1 infusion (days)	10.0 (8.0–18.0)	8.0 (6.5–10.0)	0.127
Balloon atrioseptostomy	10 (76.9)	72 (82.8)	0.699
Age at balloon atrioseptostomy (days)	1.0 (1.0–1.0)	1.0 (1.0–1.0)	0.291
Redo-balloon atrioseptostomy	0 (0.0)	4 (4.6)	1.000
Surgery prior to arterial switch	2 (15.4)	0 (0.0)	0.016
Coarctation repair before arterial switch	1 (7.7)	0 (0.0)	0.244
Cardiopulmonary resuscitation	0 (0.0)	4 (4.6)	1.000
Inotropic support	4 (30.8)	7 (8.0)	0.035
Duration of inotropic support (days)	1.0 (1.0–6.0)	1.0 (1.0–1.0)	0.207
Cardiac arrhythmia	1 (7.7)	8 (9.2)	1.000
Red blood cell transfusion	6 (46.2)	9 (10.3)	0.004
Oxygen supplementation	10 (76.9)	47 (54.0)	0.144
Duration of oxygen supplementation (days)	2.0 (1.2–5.2)	1.0 (1.0–3.0)	0.226
Respiratory support, non-invasive	10 (76.9)	65 (74.7)	1.000
Duration of respiratory support (days)	5.0 (2.3–13.0)	2.0 (1.0–4.0)	0.021
Mechanical ventilation	10 (76.9)	33 (37.9)	0.014
Duration of mechanical ventilation (days)	2.5 (2.0–7.3)	1.5 (1.0–2.8)	0.052
Pulmonary hypertension	2 (15.4)	5 (5.7)	0.225
Duration of inhaled nitric oxide treatment (days)	14.5 (11.3–17.8)	4.0 (2.0–4.0)	0.079
Caffeine	7 (53.8)	14 (16.1)	0.005
Respiratory distress syndrome	2 (15.4)	0 (0.0)	0.016
Administration of surfactant	1 (7.7)	0 (0.0)	0.130
Early onset infection, suspected	2 (15.4)	17 (19.5)	1.000
Late onset infection, suspected	3 (23.1)	1 (1.1)	0.007
Antibiotic therapy	9 (69.2)	37 (42.5)	0.083
Duration of antibiotic therapy (days)	3.0 (2.0–6.0)	3.0 (3.0–5.0)	0.657
NEC ≥ Bell Stage 2	2 (15.4)	0 (0.0)	0.016
Bronchopulmonary dysplasia	2 (15.4)	0 (0.0)	0.016
Intracranial hemorrhage	0 (0.0)	0 (0.0)	N/A
Periventricular leukomalacia	0 (0.0)	0 (0.0)	N/A
Seizures	0 (0.0)	1 (1.1)	1.000
Retinopathy of prematurity	0 (0.0)	0 (0.0)	N/A
Acute renal failure	0 (0.0)	1 (1.1)	1.000
Death prior to arterial switch operation	3 (23.1)	0 (0.0)	0.002

*IQR* interquartile range, *NEC* necrotizing enterocolitis, *NICU* neonatal intensive care unit, *N/A* not applicable

^a^The following information (no. of infants) was missing: Temperature at NICU admission = 1 in the “premature infants” group; Temperature at NICU admission = 1 in the control group

Weight at cardiac surgery was lower in preterm infants compared to term infants (2615 vs. 3420 g, *p* < 0.001), whereas age at surgery was similar in both groups (8.5 vs. 8.0 days, *p* = 0.616). Prematures received more often transfusions of RBC (80.0% vs. 41.4%, *p* = 0.040) and fresh frozen plasma (70.0% vs. 34.5%, *p* = 0.040) during the course of CPB. One term infant with intramural coronary artery and heart failure could not be weaned from CPB (Table 4).

**Table 4:** Data on cardiac surgery

Variable^a^	Premature infants (*n* = 10)	Term neonates (*n* = 87)	*p* value
	Patients (%) or median (IQR)	Patients (%) or median (IQR)	
Age at cardiac surgery (days)	8.5 (5.8–16.0)	8.0 (7.0–11.0)	0.616
Weight at surgery (g)	2615 (2452–2698)	3420 (3130–3748)	< 0.001
Aortic clamp time (min)	107 (92–118)	101 (93–115)	0.652
Cardiopulmonary bypass time (min)	275 (265–341)	263 (230–325)	0.322
Red blood cell transfusion	8 (80.0)	36 (41.4)	0.040
Fresh frozen plasma transfusion	7 (70.0)	30 (34.5)	0.040
Platelet transfusion	2 (20.0)	4 (4.7)	0.117
Intraoperative death	0 (0.0)	1 (1.1)	1.000

*IQR* interquartile range

^a^For those who received cardiac surgery (10 and 87, respectively)

Delayed chest closure was performed significantly more often in prematures than term born infants (70.0% vs. 33.7%, *p* = 0.037). Duration of postoperative ventilation was longer in prematures (175 h vs. 106 h, *p* = 0.038). The risk for venous thrombosis (40.0% vs. 4.7%, *p* = 0.004) was higher in premature infants and there was a trend toward more chylous effusions into thorax. Two out of three preterm infants with venous thrombosis of the superior vena cava had confirmed chylothorax. Although postoperative cardiac arrhythmias were observed in about 40% of both groups, no infant required pacemaker implantation. Two prematures who received postoperative ECMO therapy died during the postoperative course: One infant born at 35 0/7 weeks developed a right atrial thrombus formation and multiorgan failure following postoperative ECMO therapy. One infant born at 36 3/7 weeks with intramural coronary artery could not be weaned successfully from ECMO due to cardiac failure. One term infant died 54 days after ASO because of severe bacterial and fungal sepsis.

Overall mortality during hospitalization was significantly increased in prematures with d-TGA (38.5% vs. 2.3%, *p* < 0.001) compared to term infants. Duration of postoperative and total hospitalization were increased in preterm infants with d-TGA (Table 5).

**Table 5:** Postoperative course, co-morbidities, and outcomes

Variable^a^	Premature infants (*n* = 10)	Term neonates (*n* = 86)	*p* value
	Patients (%) or median (IQR)	Patients (%) or median (IQR)	
Arterial pH on ICU admission^b^	7.43 (7.39–7.46)	7.43 (7.39–7.47)	0.760
Arterial lactate on ICU admission (mg/dL)^b^	34.5 (22.5–43.0)	21.5 (18.0–26.0)	0.007
Maximum VIS within 24 h after surgery^b^	12.2 (8.2–19.1)	14.5 (8.4–23.0)	0.541
Lowest ScvO_2_ within 24 h after surgery (%)^b^	46.7 (35.4–55.0)	50.5 (43.3–59.1)	0.300
Extracorporeal membrane oxygenation	2 (20.0)	2 (2.3)	0.053
Delayed chest closure	7 (70.0)	29 (33.7)	0.037
Myocardial infarction	1 (10.0)	1 (1.2)	0.199
Cardiopulmonary resuscitation	2 (20.0)	6 (7.0)	0.195
Cardiac arrhythmia	4 (40.0)	35 (40.7)	1.000
Invasive mechanical ventilation (h)	175 (147–302)	106 (75–171)	0.038
Duration of inotropic support (days)	5.0 (3.5–11.3)	3.5 (2.0–5.0)	0.060
Sepsis, culture proven	0 (0.0)	2 (2.3)	1.000
Pneumonia	1 (10.0)	3 (3.5)	0.361
Pulmonary hypertension	4 (40.0)	23 (26.7)	0.460
Duration of inhaled nitric oxide treatment (h)	47 (29–94)	65 (26–100)	0.609
Acute renal failure	1 (10.0)	0 (0.0)	0.104
Chylous effusions into thorax	5 (50.0)	18 (20.9)	0.056
Chylothorax requiring surgical intervention	1 (10.0)	0 (0.0)	0.105
Diaphragm paresis	0 (0.0)	1 (1.2)	1.000
NEC ≥ Bell Stage 2	0 (0.0)	1 (1.2)	1.000
Duration of parenteral nutrition (days)	9.0 (6.5–15.3)	6.0 (4.0–8.0)	0.070
Seizures	0 (0.0)	1 (1.2)	1.000
Intracranial hemorrhage	2 (20.0)	2 (2.3)	0.053
Venous thrombosis	4 (40.0)	4 (4.7)	0.004
Red blood cell transfusion	9 (90.0)	70 (81.4)	0.686
Fresh frozen plasma transfusion	7 (70.0)	43 (50.0)	0.321
Platelet transfusion	2 (20.0)	11 (12.8)	0.621
Hemoglobin at discharge (g/dL)	12.6 (10.4–14.4)	12.8 (11.1–14.3)	0.935
Postoperative mortality	2 (20.0)	1 (1.2)	0.028

Postoperative outcome^*c*^	Premature infants (*n* = 8)	Term neonates (*n* = 85)	
Postoperative hospital stay in survivors (days)	24 (20–31)	16 (13–22)	0.006
Duration of total hospitalization in survivors (days)	35 (29–70)	26 (22–31)	0.014
Weight at discharge (g)	3 025 (2 842–3 550)	3 515 (3 260–3 860)	0.072

Mortality, overall (*n* = 100)	5 (38.5)	2 (2.3)	< 0.001

*ICU* intensive care unit; IQR, interquartile range, *NEC* necrotizing enterocolitis, *ScvO*_2_ central venous oxygen saturation, *VIS* vasoactive-inotropic score

^a^For those who survived cardiac surgery (10 and 86, respectively)

^b^The following information (no. of infants) was missing: arterial pH on ICU admission = 2, arterial lactate on ICU admission = 2, maximum VIS within 24 h after surgery = 2, lowest ScvO_2_ within 24 h after surgery = 2 in the control group

^c^For those who were discharged alive (8 and 85, respectively)

Gestational age (adjusted unit OR 0.383, 95% CI 0.179–0.821, *p* = 0.014) and presence of an intramural coronary artery (adjusted OR 620, 95% CI 4–82 373, *p* = 0.010) were the only variables independently associated with mortality in the entire cohort in multivariable logistic regression analysis (Nagelkerke’s R-squared 0.592).

## Discussion

In this retrospective study, we analyzed the impact of prematurity on postnatal and perioperative clinical management, co-morbidities, and mortality in infants with d-TGA. Our results demonstrate the requirement of more intensive care measures, as well as the appearance of more co-morbidities in the delivery room, and during the entire perinatal and postoperative period. Mortality, in particular during the preoperative period, was notably increased in preterm infants.

Perinatal management prior to corrective surgery has not been the focus of studies on preterm infants with d-TGA so far. Similar Apgar values indicate that primary fetal-to-neonatal transition during the first minutes of life is not fundamentally different in prematures, compared to term infants. However, we found a significantly higher rate of mechanical ventilation in the delivery room in prematures. Whether this difference was caused by a deterioration of the respiratory situation per se, by a more frequent elective intubation before performing balloon atrial septostomy, or both, remains unclear. Several delivery room management strategies for infants with critical CHD focus on the availability of urgent cardiac interventions such as balloon atrial septostomy [16]. Intubation and mechanical ventilation should likewise be anticipated and included in the delivery room management plan of premature infants with d-TGA.

During the preoperative period, 31% of premature infants with d-TGA required inotropic support, almost four times more than term born infants. Duration of inotropic support was short in both groups. In contrast, the study of Kim et al. reported that more than 40% of term born infants required preoperative inotropic support to augment cardiac output and maintain adequate systemic blood pressure, and inotropic support was associated with early postoperative mortality [17]. Due to the low number of patients, we found no significant association between inotropic support and morbidities. However, three premature infants developed NEC or pulmonary hypertensive crisis, thus required preoperative inotropes, and died before cardiac surgery. In children who survived the preoperative period, inotropic medication was not associated with mortality.

Almost every second preterm infant received RBC transfusions prior to ASO, compared to every tenth term born infant. Recently published recommendations on RBC transfusion in infants with CHD state that premature infants maintained on prostaglandin E1 before cardiac surgery may not tolerate anemia and require higher hemoglobin levels [18]. However, the proposed higher limit is not further specified, and premature infants with uncorrected d-TGA have not been included in transfusion trials so far. Even in premature infants without CHD, there is no consent on transfusion thresholds, and an association between RBC transfusions and neonatal morbidity or mortality is yet inconclusive [19].

Two premature infants developed NEC prior to ASO and one term born infant after cardiac surgery. Both preterm infants died shortly after diagnosis of NEC. Prematurity and presence of a complex CHD are risk factors for NEC and simultaneous occurrence of both even further increases the risk for NEC [20, 21]. In addition, RBC transfusions in term newborns with CHD are associated with development of NEC [22]. Prevention of NEC should be a major target in the management of premature infants with d-TGA and include feeding regimes and transfusion strategies. The preoperative period in preterm infants with d-TGA is highly critical and characterized by many further co-morbidities typical for prematurity, such as apnea of prematurity, respiratory distress syndrome, bronchopulmonary dysplasia and jaundice, a higher rate of suspected late onset infection, and increased mortality, in contrast to infants born at term.

Median age at cardiac surgery was similar in both groups, and weight at surgery was above 2000 g in all infants of our cohort. Recent data show that ASO can be performed safely in infants weighing as little as 2000 g and delaying repair to await further growth might not confer any benefit [11]. Higher rates of transfusion of RBC and fresh frozen plasma during CPB in preterm infants could be partly explained by hemodilution, as the ratio of CPB circuit volume to the child's blood volume becomes increasingly unfavorable with decreasing body weight. However, transfusion-free ASO has been shown to be feasible in a newborn with a body weight as little as 1700 g [23].

Although arterial lactate on postoperative admission to ICU was higher in premature than term born infants, cardiovascular support, as quantified by vasoactive-inotropic score, was comparable between both groups. However, maximum postoperative vasoactive-inotropic score was not correlated with length of hospitalization or mortality in neonates after congenital heart surgery [24]. The chest was left open liberally in infants with low body weight to mitigate hemodynamic instability. No difference was observed in the risk for acute renal failure with the requirement of dialysis. In contrast, Ahlström et al. reported a significantly increased frequency of peritoneal dialysis in preterm infants after ASO [7]. The overall incidence of postoperative renal failure in our cohort was considerably lower than previously reported rates of up to 40% in newborns with d-TGA [17]. Preterm infants had an increased risk of postoperative venous thrombosis and a trend toward higher rates of chylothorax. Reduced activity of the fibrinolytic system in prematures, as well as placement of central lines and CPB cannulas, can facilitate venous thrombus formation, which often remains undetected [25]. In addition, upper extremity vein thrombosis is associated with chylothorax development after pediatric cardiac surgery [26].

Apart from prematurity, intramural coronary artery was the only factor independently associated with overall mortality in our cohort. Previous studies investigating a potential association between intramural coronary artery and mortality report contradictory results [9, 27].

Prematurity significantly increased the risk for pre- and postoperative co-morbidities, length of hospitalization, and mortality in our cohort of newborns with d-TGA. This association of prematurity and poor postoperative outcome has been found in other studies. Cain et al. found prematurity to be the only variable associated with prolonged postoperative hospitalization in a cohort of 70 neonates with d-TGA [8]. Qamar reported that gestational age less than 36 weeks negatively impacts hospital survival of patients with d-TGA [9]. Curzon et al. reported a more than fourfold increased mortality rate in infants weighting less than 2 500 g at the time of ASO [28]. Ahlström et al. found an association between prematurity and major postoperative morbidity, prolonged mechanical ventilation, and ICU stay [7]. In contrast, Anderson et al. found no association between earlier gestational age (< 38 weeks) and morbidity or mortality in d-TGA patients. However, their study included only infants of at least 36 weeks of gestational age [6].

Early corrective cardiac surgery before clinical deterioration is generally recommended, but increases the risk for adverse outcomes in prematures [12, 15, 29]. Besides improving treatment for preterm infants with d-TGA, prenatal counseling and obstetric interventions should target at decreasing the likelihood of premature delivery for infants with prenatal diagnosis of d-TGA, as a delay in delivery might substantially improve outcomes in these high-risk patients [30].

This study has several limitations, such as its retrospective character and the single institutional focus. Due to the small number of preterm infants with d-TGA, it was not possible to further divide prematures in different subgroups depending on the gestational age. Various treatments, such as postnatal intubation or perioperative RBC transfusion, were carried out when clinically indicated by the attending physicians, without clear institutional guidelines for the treatment of premature infants with d-TGA due to a lack of evidence. It is therefore possible that practice variations occurred during the study period. The sample size and low overall rate of certain morbidities in our cohort might have precluded detection of the potential effect of prematurity on complications and morbidities. However, this is the first study to provide detailed analysis of treatment and co-morbidities between birth and corrective surgery in premature infants with d-TGA. In addition, we report on operative outcomes during the study period. Due to the high risk for complications, the management of the premature infant with d-TGA remains challenging. A multidisciplinary approach is recommended to provide optimal preoperative care in premature infants with d-TGA, including neonatologists, intensive care physicians, and pediatric cardiologists. Further research is needed to better understand factors contributing to premature delivery, to improve clinical treatment, and to investigate the impact of prematurity-related co-morbidities on long-term developmental outcomes.

## Conclusion

Physicians treating preterm infants with d-TGA should be aware of the high risk of postnatal and perioperative co-morbidities, anticipate corresponding problems, and consider appropriate treatment options at an early stage.

## Data Availability

The data that support the findings of this study are available upon reasonable request from the corresponding author [VB]. The data are not publicly available due to ethical restrictions, them containing information that could compromise research participant privacy/consent.
